# Supplementary material for: Chromosome genome assembly for the meagre, Argyrosomus regius, reveals species adaptations and sciaenid sex-related locus evolution
Source: Front Genet. 2023 Jan 10;13:1081760. doi: 10.3389/fgene.2022.1081760 (PMC9871315; doi:10.3389/fgene.2022.1081760)
Supplement: Supplementary file 4 [file DataSheet5.PDF]

Chromosome genome assembly for the meagre, *Argyrosomus regius,* reveals species adaptations and sciaenid sex-related locus evolution

Supplementary Data

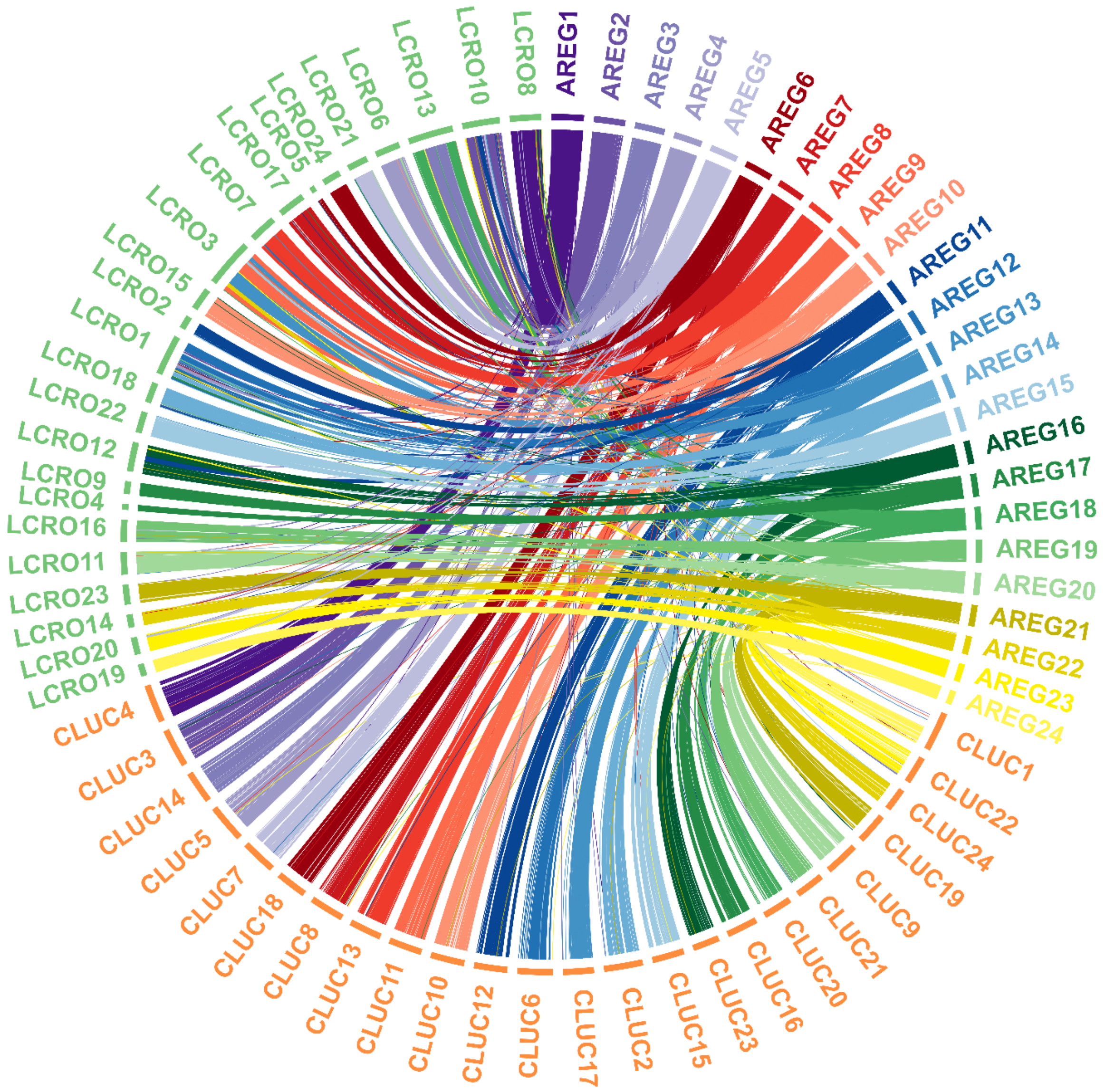

Supplementary Figure 1. Circos plot of one-to-one synteny of single copy orthologous loci in Sciaenidae genomes (CLUC: *Collichthys lucidus*, LCRO: *Larimichthys crocea*, AREG: *Argyrosomus regius* – meagre). Loci in each meagre chromosome (colour coded differently) are connected with coloured lines (corresponding to meagre chromosomal colour codes) to orthologous loci in other genomes (orange for *C. lucidus*, green for *L. crocea*). Meagre chromosomes are ordered by length. Chromosomes of *L. Crocea* and *C. lucidus* are ordered according to the order of corresponding homologous meagre chromosomes. Homologous chromosomal pairs are deduced based on the largest number of shared orthologous loci between chromosomes.

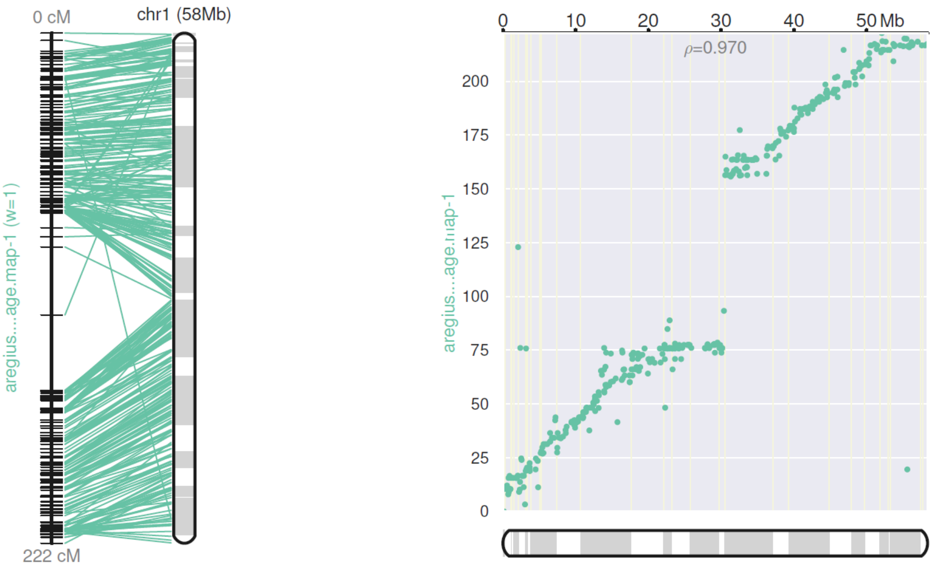

Supplementary Figure 2. Original Linkage Group I Mapping to Assembly Contigs. Scaffolding via ALLMAPS revealed assembly contigs mapped to two non-overlapping regions of the original Linkage group I of the meagre linkage map, suggesting the linkage group was a merge of two constituent groups.

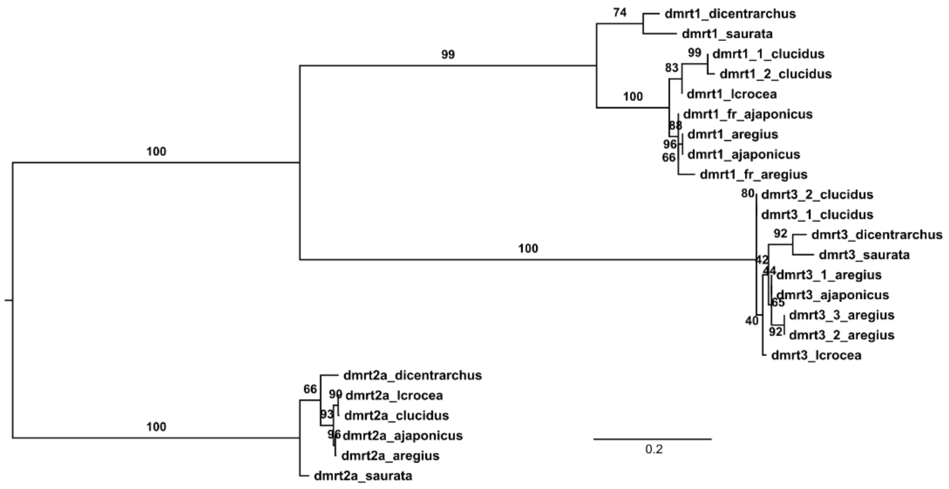

Supplementary Figure 3. Phylogenomic reconstruction of sciaenid DMRT1, DMRT2 and DMRT3 protein relationships. DMRT protein sequences from each species were aligned using MAFFT, followed by trimming with trimAl (gap threshold of 20%). Tree inference was carried out with RAxML-ng, using the JTT model (selected by ModelTest-NG) and bootstrap resampling with 100 bootstrap replicates.

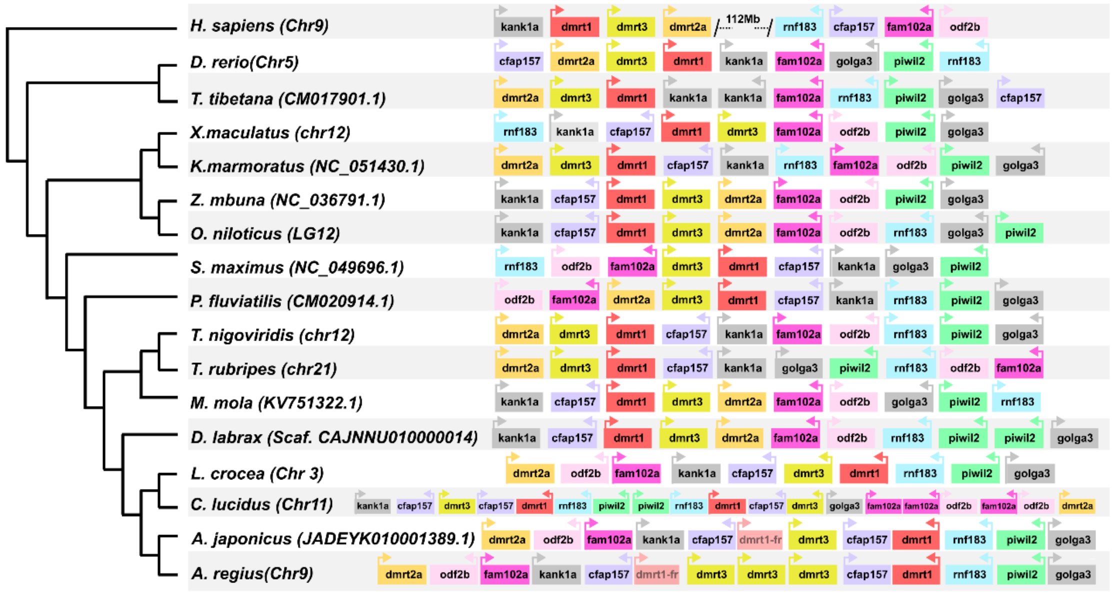

Supplementary Figure 4. Extended synteny diagram for *dmrt1* neighbourhood genes in different teleosts and human. The chromosome or scaffold of the neighbourhood in each species is given next to the species name. Genes are colour coded by gene name, following the same code as in main Figure 6. Relative orientation (strand information) is indicated by coloured arrows in each box.

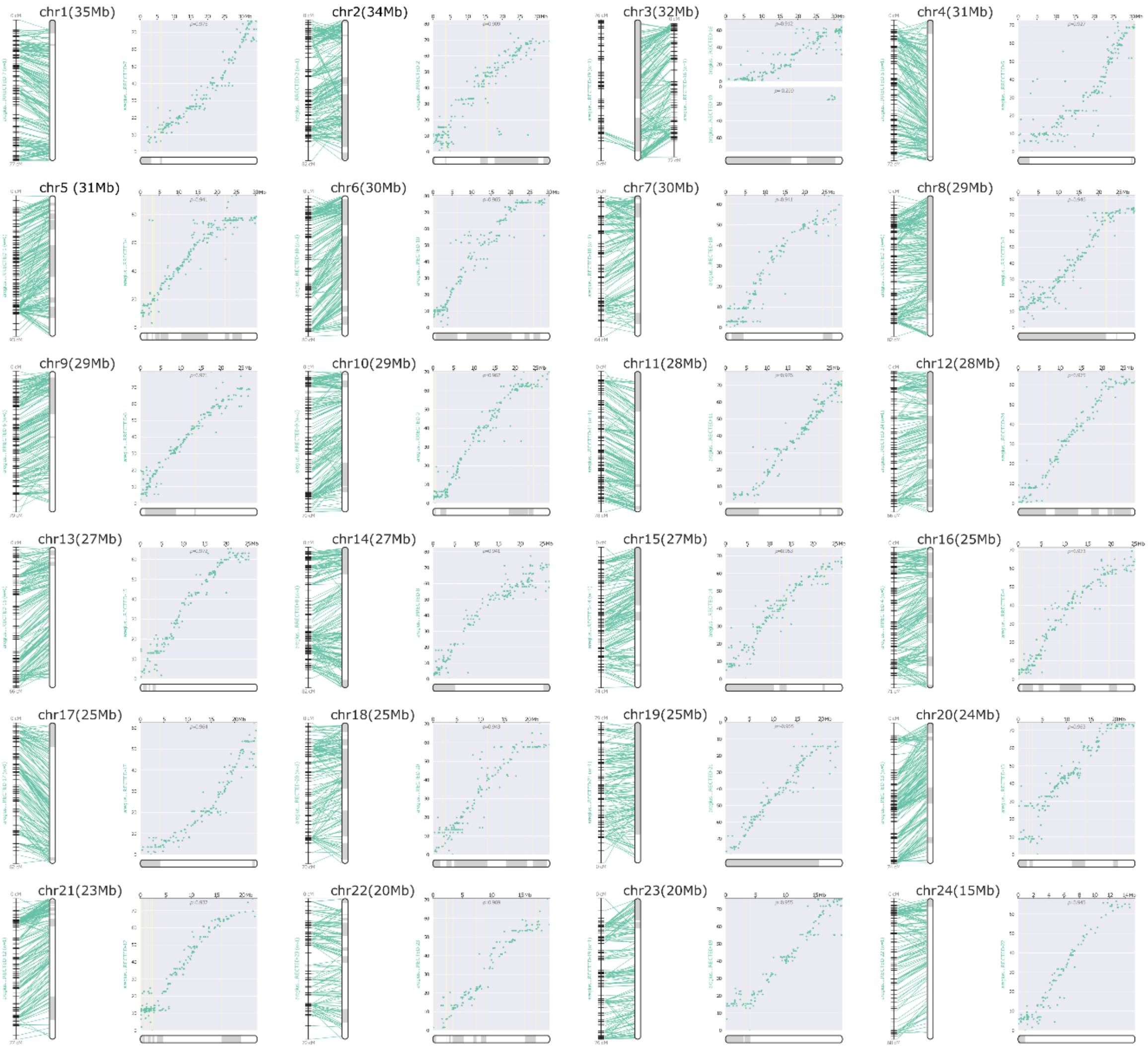

Supplementary Figure 5. ALLMAPS scaffolding result mapping plots for each reconstructed chromosomal scaffold. Each plot includes two subplots, with both presenting the same mapping. The left subplot presents the mapping of linkage map markers (vertical lines on the left of the subplot) to assembly contigs included in each scaffold (grey/white regions in bar on the right of the sublot) by drawing connecting lines between the two. The right subplot plots linkage map marker coordinates (y axis) against scaffold coordinates on the x axis.

Supplementary table 1. Genome sequencing quality statistics.

Supplementary table 2. Transcriptome sequencing quality statistics.

Supplementary table 3. ENSEMBL proteomes used for homology evidence during annotation.

Supplementary table 4. Genomes used for comparative genomics analyses.

Supplementary table 5. Meagre OrthoFinder Hierarchical OrthoGroups (HOGs) with duplications. These HOGs were selected through a comparison of CAFE and GeneRax output, as described in Materials and Methods.

Supplementary table 6. GO enrichment analysis via gprofiler on duplication containing orthogroups from all species. Ontology terms (GO biological) significantly enriched (adjusted p value <= 0.1) in meagre duplications are found on the top of the list, ranked by the number of other species in which each term is also significantly enriched.

Supplementary table 7. Fast evolving transcript phyloP (CONACC) scores.

Supplementary table 8. Slow evolving transcripts phyloP (CONACC) scores.

Supplementary table 9. Analysis of phyloP score for meagre genes in duplication containing orthogroups.

Supplementary table 10. GO enrichment analysis via gprofiler on fast evolving genes (phyloP score <0).

Supplementary table 11. Orthofinder orthogroups (HOGs) used in the study.

Supplementary table 12. Genomic location and strand (orientation) information for genes shown in Fig.6 and supplementary Fig.4.

Supplementary table 13. MinION read mapping (via BLAST) on meagre genome assembly region chr9:22256718-22408507, containing the DMRT gene neighbourhood shown in Fig.6 and supplementary Fig.4. The whole region is covered by overlapping large reads. The region is covered by a single pair of overlapping reads (Reads c250bbb4-5e7d-479b-88cc-635b78ec788a & adc256b3-9fcb-4a22-a9bb-0b08820a8828 highlighted in yellow). A single (e31740e2-4df5-4e3f-8dad-7a916fc6d61c highlighted in red) contains all three dmrt3 copies.

Supplementary table 14. PacBio reads (SRR8142901) that map uniquely (via BLAST and LAST) on *C. lucidus* DMRT neighbourhood loci (shown in Fig.6 and

supplementary Fig.4). Reads included map uniquely on a single copy of studied loci, not matching the region of other copies.

Supplementary table 15. Linkage map scaffolding summary (ALLMAPS).
